# Plant Kinesin-Like Calmodulin Binding Protein Employs Its Regulatory Domain for Dimerization

**DOI:** 10.1371/journal.pone.0066669

**Published:** 2013-06-21

**Authors:** Maia V. Vinogradova, Galina G. Malanina, Joshua S. Waitzman, Sarah E. Rice, Robert J. Fletterick

**Affiliations:** 1 Department of Biochemistry and Biophysics, University of California San Francisco, San Francisco, California, United States of America; 2 Department of Cell and Molecular Biology, Northwestern University, Chicago, Illinois, United States of America; University of Leeds, United Kingdom

## Abstract

Kinesin-like calmodulin binding protein (KCBP), a Kinesin-14 family motor protein, is involved in the structural organization of microtubules during mitosis and trichome morphogenesis in plants. The molecular mechanism of microtubule bundling by KCBP remains unknown. KCBP binding to microtubules is regulated by Ca^2+^-binding proteins that recognize its C-terminal regulatory domain. In this work, we have discovered a new function of the regulatory domain. We present a crystal structure of an *Arabidopsis* KCBP fragment showing that the C-terminal regulatory domain forms a dimerization interface for KCBP. This dimerization site is distinct from the dimerization interface within the N-terminal domain. Side chains of hydrophobic residues of the calmodulin binding helix of the regulatory domain form the C-terminal dimerization interface. Biochemical experiments show that another segment of the regulatory domain located beyond the dimerization interface, its negatively charged coil, is unexpectedly and absolutely required to stabilize the dimers. The strong microtubule bundling properties of KCBP are unaffected by deletion of the C-terminal regulatory domain. The slow minus-end directed motility of KCBP is also unchanged *in vitro*. Although the C-terminal domain is not essential for microtubule bundling, we suggest that KCBP may use its two independent dimerization interfaces to support different types of bundled microtubule structures in cells. Two distinct dimerization sites may provide a mechanism for microtubule rearrangement in response to Ca^2+^ signaling since Ca^2+^- binding proteins can disengage KCBP dimers dependent on its C-terminal dimerization interface.

## Introduction

Kinesin-like calmodulin binding protein (KCBP) is a molecular motor found in plants [1]. KCBP is active during different stages of mitosis [2], [3]. However, its activation and silencing is crucial mainly for normal trichome morphogenesis [4]. Both mitosis and trichome morphogenesis, though discrete processes, rely on correct cytoskeleton structure, which is based on microtubules and actin filaments. *In vitro*, active KCBP promotes formation of microtubule bundles while its negative regulation promotes dissociation of microtubule bundles [5].

**Figure 1:**
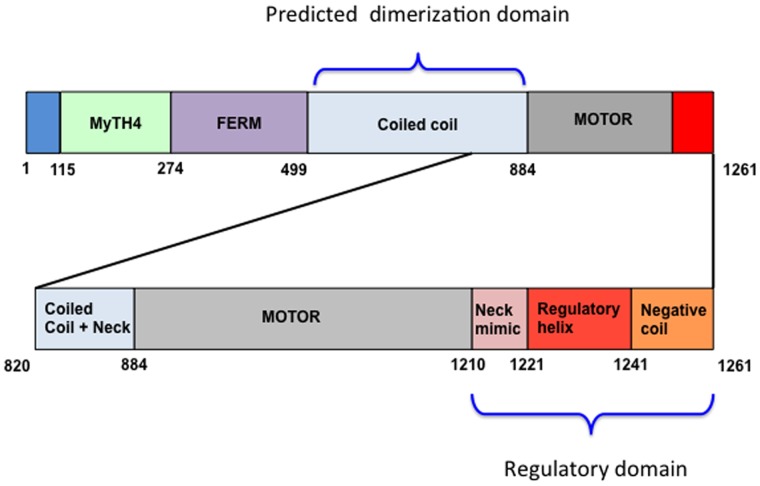
Schematic presentation of the domain organization in KCBP.

KCBP belongs to the kinesin family of molecular motors. Molecular motors of this family use the energy of ATP hydrolysis to drive a mechanical power stroke, leading to their directional movement along microtubules [6]. KCBP has a typical kinesin motor domain often referred to as a head. This domain attaches to microtubules and contains a functional nucleotide-binding site. However, KCBP has an unusual N-terminal tail domain that relates KCBP to another family of molecular motors, myosins, which move along actin filaments. Just like the tails of myosins VIIa and X, the tail of KCBP contains talin-like FERM domains and MyTH4 homology regions with additional affinity to microtubules [7] (Fig. 1).

The motor head of KCBP is found near the C-terminus of its polypeptide chain. This structural organization places KCBP in the Kinesin-14 group of the kinesin family, together with its structural relatives, Drosophila ncd, yeast KAR3, and others [8]. Molecular motors of the Kinesin-14 group move toward the minus end of the microtubule, which has alpha subunits of tubulin exposed. KCBP has been reported to move at ∼8 µm/min [9], a velocity comparable to that of ncd (∼10 µm/min) [10]. A coiled coil is predicted to form functional dimers of KCBP (Fig. 1) using a segment a.a. 749–855. This dimerization domain precedes the motor head within the protein sequence [11].

KCBP has another unusual structural domain that distinguishes it among kinesins, at the very C-terminus of the polypeptide chain. The C-terminal regulatory domain of KCBP consists of three structural features coil-helix-coil. These features are termed the neck mimic, regulatory helix, and negative coil, respectively [12]. Two of these features, the regulatory helix and the neck mimic, have been previously characterized. The regulatory helix is recognized independently by calmodulin and additionally by a specific KCBP regulator, the Ca^2+^ ion sensor KIC [13]. KIC is a specialized calmodulin with just two Ca^2+^ ion coordinating EF hands, one of them being disabled by mutations, instead of four EF hands present in calmodulin. When bound to KCBP, these Ca^2+^-binding proteins cause the motor to detach from microtubules and thus negatively regulate its activity. The neck mimic both links the regulatory helix to the motor domain and plays an important role in the conformational response to nucleotide binding. The neck mimic docks along the motor domain, stabilizing the ATP-bound conformation of KCBP [12]. KIC captures the neck mimic in the presence of calcium, preventing it from docking and thus precluding KCBP from responding normally to ATP binding [14].

However, the role of the third feature of the regulatory domain, the negative coil, in the function of KCBP is unclear. One possible role of the negative coil that was proposed [14] is to electrostatically destabilize the interactions between the KCBP-KIC complex and the negatively charged surface of the microtubule. The electrostatic repulsion would be in addition to a major steric conflict introduced by KIC [14] and would facilitate the detachment of the motor-regulator complex from microtubules. However, in the structure of the unregulated motor, KCBP alone [12], the negative coil is positioned between the motor and the microtubule surface and thus would destabilize microtubule binding of the unregulated motor as well.

To determine the role of the negative coil in the function and regulation of KCBP, we initiated a series of structural and biochemical experiments. These experiments steered us to the discovery of a previously unrecognized feature of the *Arabidopsis* KCBP regulatory domain, namely, that it can self-associate, leading to a dimerization of *Arabidopsis* KCBP through its C-terminus. Here we present structural and biochemical data showing that the *Arabidopsis* KCBP dimers formed via association of the C-terminal regulatory domains exist both in crystals and in solution and that the negative coil is indispensable for maintaining dimerization of KCBP at its C-terminus. To address the physiological relevance of this unpredicted dimerization, we expressed the constructs of *Arabidopsis* KCBP with and without C-terminal regulatory domain and compared their biological properties in motility and microtubule bundling assays. Although the self-association of the C-terminal regulatory domain did not affect the biological function of KCBP in these assays, the geometry of the dimer structure suggest to us that KCBP may engage this feature to support Ca ion-dependent specific microtubule-based structures in cell.

## Materials and Methods

### Expression Constructs of KCBP and KIC

The *Arabidopsis* DNA constructs of KCBP (12–1261), KCBP (876–1261), KCBP (884–1253), and KCBP (884–1244) were cloned in pET28b using NcoI-EcoRI sites (generously provided by A.S.N.Reddy). The C1130N mutation was inserted in KCBP (876–1261) construct using QuikChange site-directed mutagenesis kit (Stratagene). All the pET28b KCBP constructs encoded a tag-free protein.

The *Arabidopsis* DNA construct of KCBP (820–1225) was cloned into the vector pET32 Xa/Lic (Novagen) using the kit and protocols for ligation independent cloning (LIC). The plasmid encoded the N-terminal His_6_-Trx tag separated from the expression gene by a linker with the TEV-protease cleavage site.

The *Arabidopsis* DNA construct of KCBP (884–1225) was cloned into pDEST17 (Invitrogen) using the kits and protocols for GATEWAY cloning technology. The forward PCR primer used for cloning was designed to insert the TEV-protease cleavage site between N-terminal His_6_ tag and the expression gene.

The full length *Arabidopsis* KIC (1–135) was cloned into a modified pRSFduet plasmid (Novagen) adapted for Gateway cloning technology (Invitrogen) encoding the N-terminal His_6_ tag cleavable by TEV-protease.

The *Arabidopsis* KIC (29–135) was cloned into the vector pET32 Xa/Lic (Novagen) using the kit and protocols for ligation independent cloning (LIC). The plasmid encoded the N-terminal His_6_-TRX tag separated from the expression gene by a linker with the TEV-protease cleavage site.

### Protein Expression and Purification

For protein expression the described constructs were transformed into E. coli competent cells BL21(DE3). The cells were allowed to grow at 37°C until OD_600_ ∼0.6–0.8. Protein expression was induced by adding 0.1 mM IPTG to the cell culture. After 3–16 h of expression at 25°C, the cells were harvested. The cell pellets containing the recombinant KCBP or KIC were subjected to lysis by sonication in the buffer containing 50 mM Tris (pH7.5), 50 mM NaCl, 2 mM MgCl_2_, 2 mM CaCl_2_, 0.1 mM ATP, 1 mM TCEP, and protease inhibitors mixture. The recombinant proteins carrying the His_6_-tag were purified from the soluble fraction of the cell lysate using the Ni-NTA beads (Amersham). The Ni-NTA bound proteins were eluted in the presence of 100 mM imidazole. To cut the tag peptide off, the protein samples were treated with TEV-protease while dialyzed overnight against the original imidazole-free buffer. Then, the sample was passed through the Ni-NTA beads again. The unbound fraction containing the tag-free protein was collected. The KCBP proteins expressed carrying no tag were purified out of the soluble fraction of the cell lysate using Calmodulin-Sepharose 4B (Amersham) as described in [12].

### Gel-filtration

Size-exclusion chromatography was done using Superdex 200 16/60 column (Amersham) and the AKTA chromatography system (GE biotech). The gel-filtration buffer contained 50 mM Tris (pH7.5), 150 mM NaCl, 2 mM MgCl_2_, 0.1 mM ATP, 1 mM TCEP, and either 1 mM EGTA or 2 mM CaCl_2_.

### Crystallization, Data Collection, and X-ray Structure Determination

Before crystallization, the Arabidopsis ^C1130N^KCBP (876–1261) was purified using Calmodulin-Sepharose 4B and concentrated up to 10–15 mg/ml. Crystals were grown by using the vapor-diffusion method, in sitting drops under the following conditions: 10% PEG 3000, 100 mM imidazole (pH 8.0), 200 mM Li_2_SO_4_, at +4°C. Before data collection, the crystals were frozen in liquid nitrogen. 15% ethylene glycol was used as a cryo-protectant. Data collection was done at the Advanced Light Source (Lawrence Berkeley National Laboratory, Berkeley, CA) Beamline 8.3.1 (λ = 1.1 Å) by using a single crystal. Data were integrated and scaled by using HKL2000 software package. The crystal was of the primitive monoclinic space group P2_1_ with cell dimensions a = 45.7 Å, b = 75.1 Å and c = 120.6 Å, and α = 90°, β = 91.45°, γ = 90°. The asymmetric unit contained 2 molecules of KCBP. The solvent fraction of the crystal is 46.9%, and the Matthews coefficient is 2.4 A^3^/Da. The structure of the *Arabidopsis* ^C1130N^KCBP (876–1261) was determined by molecular replacement using CNS software [15], the atomic coordinates for the previously solved structure of potato KCBP motor domain (PDB ID 1SDM) as a search model. The model was built and refined using the CNS software alternating with manual rebuilding steps using Coot. Scaling and refinement statistics are detailed in Table 1. Each molecule of KCBP in the asymmetric unit was built and refined independently. The final model contained two polypeptide chains of KCBP, A and B. Chain A comprised residues 886–1124 and 1137–1253 of *Arabidopsis* KCBP, chain B comprised residues 877–1077, 1085–1122, 1139–1239, and 1244–1252 of *Arabidopsis* KCBP. Two Mg^2+^-ADP, 98 water molecules, 4 molecules of imidazole, and 3 molecules of ethylene glycol were also modeled into visible electron density. The final model was submitted to PDB database (PDB ID 4FRZ).

**Table 1:** Data collection and model refinement statistics.

Space group	P2_1_
Unit cell	a = 45.7 Å, b = 75.1 Å, c = 120.6 Å,α = 90°, β = 91.45°, γ = 90°
Molecules per asymmetric unit	2
***Data collection***	
Resolution range (Å)	25.00–2.40
Highest resolution shell (Å)	2.49–2.40
Observed reflections	235558
Unique reflections	29494
Completeness (%)	91.9 (81.9)*
Redundancy	2.6 (2.0)*
I/σ(I)	11.5 (2.4)*
R_sym_ (%)	8.3 (26.1)*
***Refinement***	
Resolution range (Å)	25.0–2.4
R_cryst_ (%)	22.5
R_free_ (%)	26.3
R.m.s deviation from ideality	
Bonds (Å)	0.011
Angles (°)	1.63
Average B-factor (Å^2^)	28.75

* Numbers in parentheses are given for reflections in the highest resolution shell.

### Analytical Ultracentrifugation

Analytical ultracentrifugation was done according to the standard manufacturer protocol for the method of sedimentation equilibrium using Beckman Optima XL-A. Purified recombinant KCBP (884–1253) and KCBP (884–1244) at three different concentrations (OD_280_ ∼0.25, OD_280_ ∼0.4, OD_280_ ∼0.5) were subjected to spin at 5 different speeds ranging between 3,000 rpm and 16,000 rpm. The sedimentation curves were registered at 281 nm, 5.8°C, and processed using UltraScan 3.0.

### Differential Interference Contrast Microscopy

Taxol-stabilized microtubules were prepared as described in [16]. The microtubules-KCBP complexes were assembled in the buffer containing 20 mM PIPES, pH 6.9, 200 mM NaCl, 2 mM MgCl_2_, 1 mM DTT, 20 µM taxol, and either no nucleotide or 5 mM ATP or ADP, or AMPPNP. The concentration of microtubules (tubulin dimers) was varied in the mixtures from 5 to 10 µM. KCBP (884–1253) or KCBP (884–1225) were added to the final concentration of 1–10 µM to achieve various molar ratios of KCBP *vs* tubulin from 1∶10 to 1∶1. When needed, KIC was added to the protein mixtures at the molar ratio of 1∶1 to KCBP and the buffer was then supplemented with 2 mM CaCl_2_. The samples were prepared at room temperature and 5 µl of the prepared sample was immediately placed between two glass cover slips and sealed to prevent evaporation. The sample was then analyzed using differential interference contrast microscopy.

### MT-gliding Assay

KCBP purified as described above was thawed, combined with an excess of porcine microtubules and 1 mM AMPPNP, and centrifuged at 100,000×g for 15 minutes at room temperature. 5 mM ATP and 200 mM NaCl was added to the resulting pellet to release the active fraction of KCBP and the mixture was centrifuged at 100,000×g for 10 minutes. Active KCBP was present in the supernatant. Motility analysis was performed with an ATP regenerating system and oxygen scavengers as described in [17] in buffer containing 50 mM Tris pH 7.5, 2 mM MgCl2, 1 mM EGTA, 50 mM NaCl, 1 mM DTT, 1 mM ATP and 0.2 mg/mL BSA. Briefly, motors were attached to the surface of a flow cell via an anti-His antibody (AbCam H8); polarity-labeled microtubules (with their minus ends bright) and ATP were added, and microtubule-positions was observed every 10 seconds for 10 minutes. Microtubules were tracked using ImageJ, and velocities were calculated for every point along their tracks.

## Results

### Ordering of an Entire Regulatory Domain of KCBP in Crystals

To clarify the function of the negative coil in the structure of KCBP, we performed crystallographic studies to better characterize this element on a structural level.

**Figure 2:**
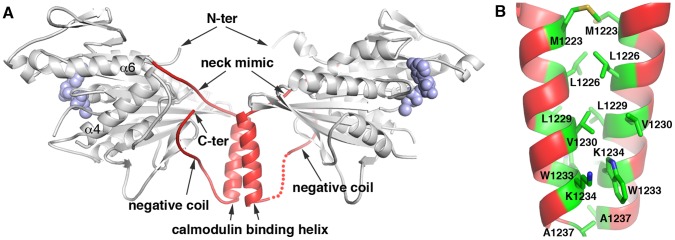
Dimer organization of KCBP. A- Crystal structure of *Arabidopsis* KCBP solved at resolution of 2.4 Å. Two molecules of KCBP (a.a. 876–1261) found in the crystal asymmetric unit are shown. Structural elements are presented as cartoon. ADP and Mg ions are shown as space-filling model in blue. Motor domain is in grey. The regulatory domain is highlighted in red. The unstructured fragment of the regulatory domain in one of the molecules is shown in dotted line. The structural elements of the regulatory domain and N- and C-termini are indicated. Helices α4 and α6 are indicated for the viewer orientation. B- Close-up presentation of the dimer interface made of the regulatory helices. The interacting residues are shown as sticks and indicated. Color-coding is by element: carbonyls in green, nitrogens in blue, sulphur in yellow.

In a previous X-ray crystal structure of the KCBP motor domain (a.a. 884–1252) from *Solanum tuberosum* (potato) [12], [18], the negative coil interacted with the microtubule-binding surface of KCBP. However, the fragment of the polypeptide chain connecting the negative coil and the regulatory helix was not visible due to the lack of order and, therefore, was missing in these structures. Missing residues made interpretation of the structural data uncertain, as the negative coil observed interacting with a KCBP head could either belong to the same molecule or could belong to a neighboring molecule in the crystal. To determine whether the negative coil belongs to the same molecule or is a swapped domain, we crystallized the KCBP motor domain (a.a. 876–1261) from *Arabidopsis* and obtained a different crystal lattice of P2_1_ space group, with 2 KCBP molecules per asymmetric unit. For one of the two molecules, the regulatory domain was visible through its entire length (Fig. 2a). The link between the regulatory helix and the negative coil was modeled unambiguously into visible electron density. Our model indicates that the domain swap does not play a role in positioning of the negative coil over the microtubule-binding surface of KCBP in the *Arabidopsis* KCBP crystals. The observed conformation of the negative coil is allowed solely by the folding of one polypeptide chain, without a domain swap.

The N-termini of two molecules display different degrees of order. One molecule in asymmetric unit has a short coil at the N-terminus while nine additional amino acids of the N-terminus in the second molecule are observed as a short α-helix, a fragment of the predicted helical neck domain. The differences in the structures of both N-and C-termini in two molecules of KCBP surely relate to the different environments in the crystal lattice.

### KCBP Forms a Dimer in Crystals

**Figure 3:**
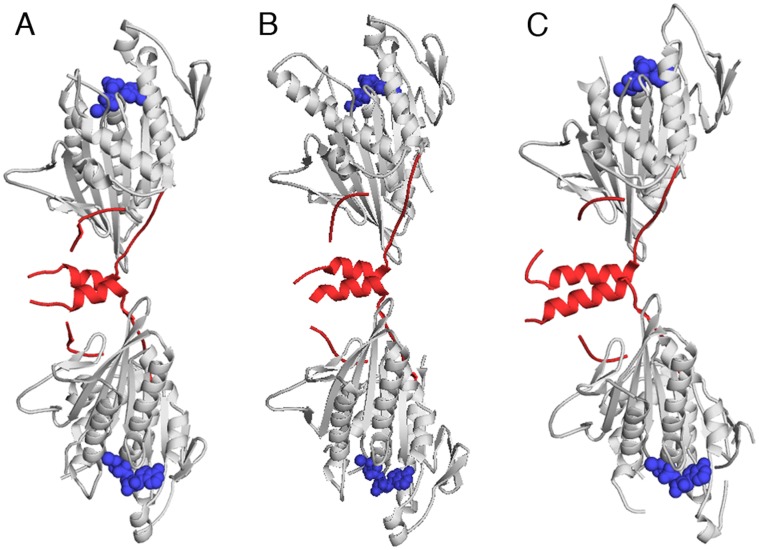
Dimeric assemblies in the crystals of potato KCBP. Crystal structures of potato KCBP solved at resolution of *A-* 2.3 Å, PDB code 1SDM.pdb; *B-* 2.2 Å, PDB code 3COB.pdb; *C-* 2.9 Å, PDB code 3CNZ.pdb. Structural elements of KCBP are shown in cartoon model. Motor core is in grey. Regulatory domain is highlighted in red. ADP is shown as a space-filling model in blue.

A prominent feature of the two molecules of KCBP in the asymmetric unit of the *Arabidopsis* KCBP crystals is that they interact with each other via the regulatory helices (Fig. 2a). These interactions form from the hydrophobic residues of the regulatory helices and cover about 630 Å^2^ on each molecule (Fig. 2b). The calculated free energy gain, assuming no structural changes, from the dimerization was −13.8 kcal/mol (PDB ePISA). To find out whether a similar arrangement is found in the crystals of potato KCBP, we analyzed the previously solved structures (Fig. 3). We found that in crystal structures of potato KCBP the regulatory helix always interacted with its counterpart from either a molecule related by crystallographic symmetry (structure 1SDM.pdb, Fig. 3A) or a second molecule in the crystal asymmetric unit (structures 3COB.pdb and 3CNZ.pdb, Fig. 3B and Fig. 3C). These interfaces were not highlighted since the interfaces are less extensive, however, the nature of the interactions supporting dimerization via the regulatory helices was always hydrophobic. Thus, in crystals, two orthologs of KCBP form dimers via the hydrophobic interactions between the regulatory helices of each monomer.

There are two microtubule-binding surfaces for each dimer of KCBP. The two microtubule-binding surfaces in the dimer are oriented such that bound microtubules would be orthogonal.

### The Regulatory Helix Enables KCBP to Form Dimers in Solution

**Figure 4:**
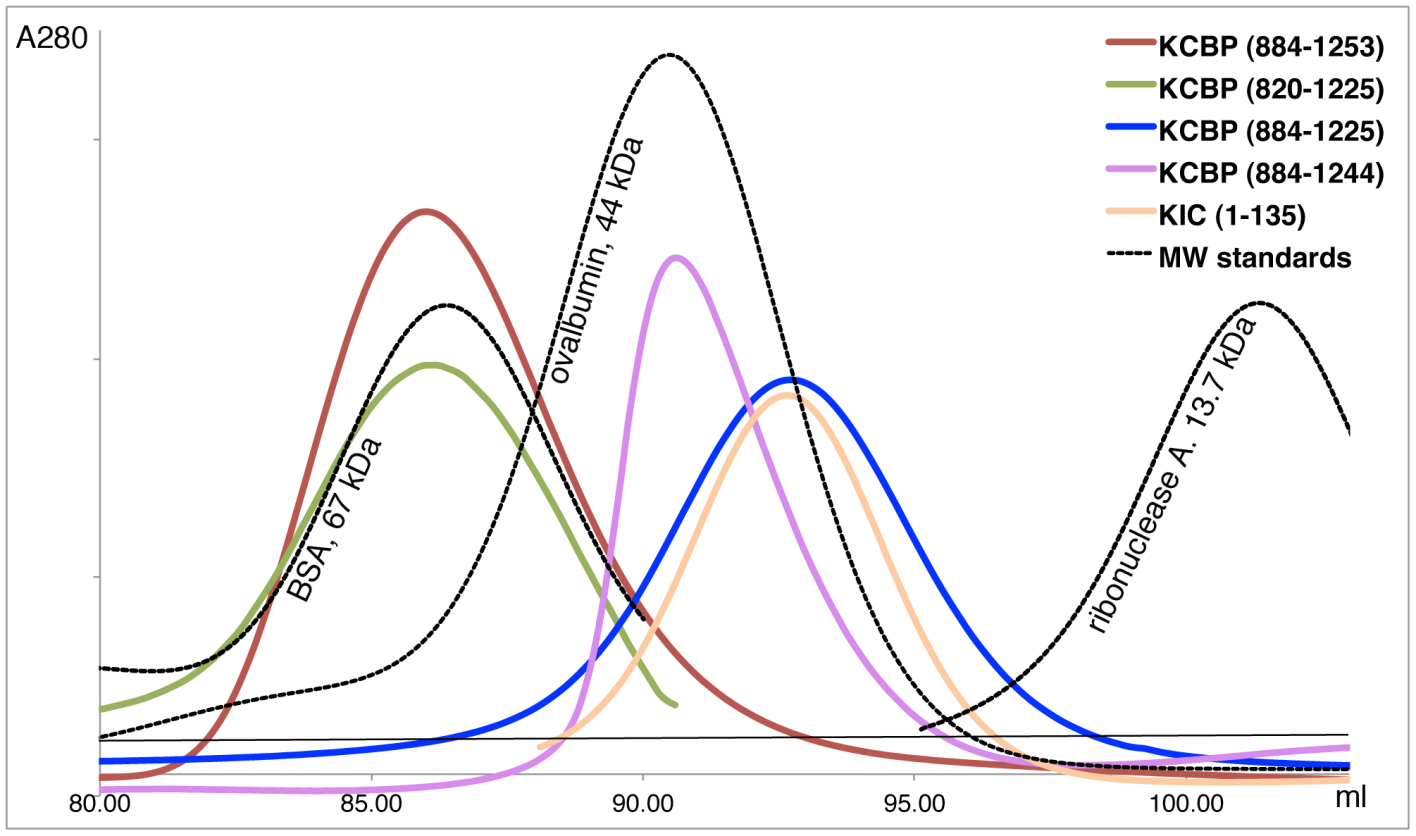
Gel-filtration profiles of the isolated KCBP constructs on Superdex200Profiles corresponding to the different KCBP samples are color coded and indicated. Profiles of the protein standards are shown as dashed lines in black.

To determine whether dimerization of KCBP takes place in solution, we prepared a truncated construct of *Arabidopsis* KCBP (a.a. 884–1225), lacking the regulatory helix and the negative coil. Then, we compared the *Arabidopsis* KCBP (a.a. 884–1253) with an intact regulatory helix and the truncated KCBP using size exclusion chromatography (Fig. 4). We observed that the molecular weight of the truncated construct was 2-fold less than the molecular weight of the KCBP construct with an intact regulatory helix (Table 2). The calculated values of the molecular weight for KCBP and the truncated KCBP were 72 kDa and 35 kDa, respectively. These values were lower than the predicted values of 84 kDa and 42 kDa. Nonetheless, our findings indicate that KCBP forms stable dimers in solution and that the C-terminal peptide encompassing the regulatory helix and the negative coil enables dimerization.

**Table 2:** Molecular weight of different KCBP and KIC constructs determined by gel-filtration on Superdex 200.

Protein	Predicted MW	Measured MW
KCBP (884–1253)	42 kDa	72±2 kDa
6-His-KCBP (884–1225)	42 kDa	36±2 kDa
KCBP (884–1244)	41 kDa	41.4±0.5 kDa
KCBP (820–1225)	46 kDa	72±1 kDa
KIC (1–135), Egta or Ca^2+^	15 kDa	34±1 kDa
KIC (29–135), Egta or Ca^2+^	12 kDa	24±1 kDa

### Deletion of the Negative Coil Abolishes Dimerization via Regulatory Helix

The interface supporting the observed dimerization of KCBP via the regulatory helix does not seem extensive enough to produce a stable dimer in solution. To determine whether the negative coil contributes to stability of the observed dimers, we prepared truncated KCBP (884–1244) lacking most of the negatively charged amino acids at the C-terminus but long enough, by 3 amino acids, to support the helical conformation of the preceding residues of the regulatory helix. The molecular weight of this truncated construct was compared with the molecular weight of KCBP (884–1261) with the intact regulatory domain by analytical ultracentrifugation using the method of sedimentation equilibrium. We found that a 1-component model best described sedimentation equilibrium for both constructs (Figure S1). The molecular weight for KCBP (884–1261) was estimated to be 89±1 kDa and corresponded to a dimer. The molecular weight for KCBP (884–1244) was estimated to be 49±1 kDa and corresponded to a monomer. This molecular weight of KCBP (884–1244) is close to the molecular weight estimated from gel-filtration (41 kDa, Table 2). These findings indicate that regulatory domain of KCBP is engaged in dimerization and that the negative coil strengthens dimerization dramatically.

### KIC Forms a Dimer in Solution

The observed dimerization of KCBP led us to investigate the oligomeric state of its regulator KIC. When we analyzed the gel-filtration profile of KIC, we found that the calculated molecular weight for KIC, 35 kDa, was double of its predicted value (17 kDa) (Fig. 4, Table 2). The molecular weight of KIC estimated by this method was affected neither by presence/absence of Ca^2+^ nor by deletion of the first 35 N-terminal residues (Table 2). Our findings indicate that KIC also forms dimers in solution and that dimerization of KIC is not Ca^2+^-dependent and does not require the N-terminal peptide. Extensive crystallizations of KIC dimers alone were not successful. In the absence of structural data on KIC by itself, we cannot conclude whether the KIC dimer is compatible with the dimer of KCBP formed by the regulatory helices.

### KCBP-KIC Complex does not Form on Gel-filtration Column

In contrast to the stable dimers of individual KCBP and KIC, their heterodimer was not stable in solution enough to stay together during the gel-filtration. In the presence of Ca^2+^, the KCBP-KIC complex, pre-formed and isolated using binding of the His-tagged KIC on Ni-NTA agarose, dissociated on Superdex-200 into the individual homodimers.

### Deletion of Regulatory Helix does not Block the Microtubule Bundling by KCBP

**Figure 5:**
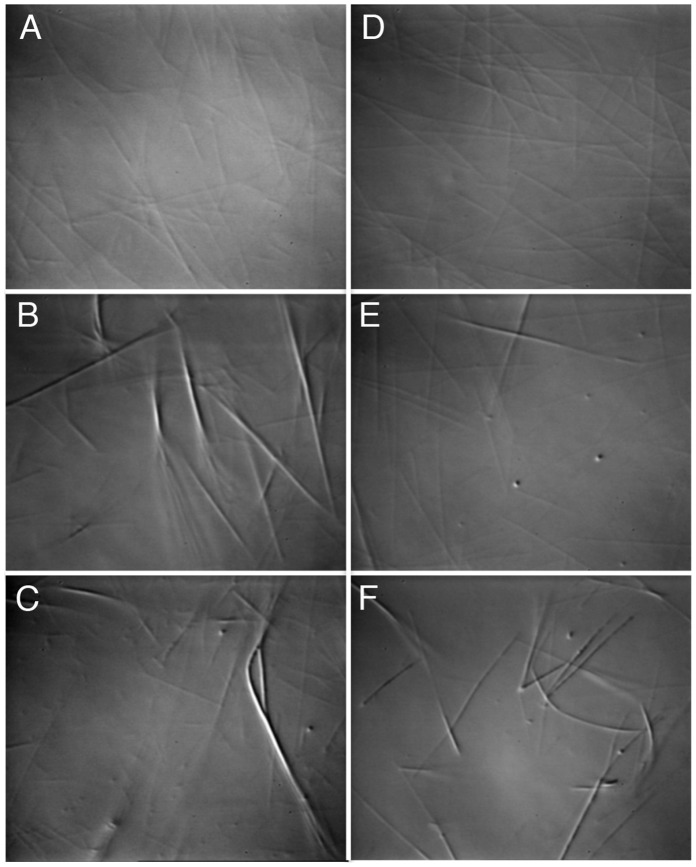
Differential interference contrast microscopy of microtubules in the presence of different constructs of KCBP. *A-* microtubules alone; *B-* microtubules plus KCBP (884–1253); *C-* microtubules plus KCBP (884–1225); *D-* microtubules plus Ca^2+^-KIC; *E-* microtubules plus KCBP (884–1253) and Ca^2+^-KIC; *F*- microtubules plus KCBP (884–1225) and Ca^2+^-KIC.

To determine the effects of the dimerization via the regulatory helix on microtubule bundling properties of KCBP, we performed microtubule bundling assays in the presence of either KCBP (884–1253) or truncated KCBP (884–1225). Microtubule bundling was observed using differential interference contrast microscopy (DIC) (Fig. 5). Both KCBP (884–1253) and KCBP (884–1225) promoted formation of microtubule bundles at concentrations as low as 1 µM and at molar ratios of kinesin to tubulin as low as 1∶10. Addition of Ca^2+^-KIC reversed the action of KCBP (884–1253), but not of KCBP (884–1225) which lacks the calmodulin/KIC-binding domain. Our results showed that the truncated construct of KCBP was sufficient to promote formation of the microtubule bundles. Thus, the regulatory domain and its self-associative properties are not required for the microtubule bundling by KCBP.

### Effect of Regulatory Domain on Motility of KCBP

To determine whether dimerization via the regulatory helix changes motility of KCBP, we performed motility assays using different constructs of KCBP. We used a construct of KCBP (a.a. 876–1261) containing the C-terminal regulatory domain but without the predicted dimerization domain at the N-terminus. We also prepared a construct of KCBP (a.a. 820–1225) with the predicted dimerization motif at the N-terminus but without the C-terminal regulatory helix. KCBP (a.a. 820–1225) eluted on gel-filtration as a dimer and its MW was measured as 72±1 kDa (Table 2).

The motility of KCBP (a.a. 876–1261) without the dimerization domain at the N-terminus could best be described as a back-and-forth movement (Movie S1). This motor appears capable of binding microtubules and taking several steps in one direction before reversing and taking several steps in the opposite direction. All microtubules in these samples showed this robust back-and-forth behavior, suggesting that this effect is not due to insufficient blocking of the glass surface. Further analysis did not demonstrate a net plus or minus end bias to these movements. Thus, the neck mimic present in KCBP (876–1261) does not act as a determinant of the directionality, unlike the true necks in the kinesins [21], and the C-terminal dimers lack directed motility.

The N-terminal dimer of KCBP without the regulatory helix, KCBP (820–1225), moved toward the minus end of the microtubules (Movie S2). Walking occurred in bursts between pauses resulting in average velocity of 3±1.5 µm/min. The large standard error in this measured velocity accurately reflects the stop-and-go kind of motility we observed. Given the differences in conditions and constructs for these assays, the velocity of 3±1.5 µm/min is reasonably close to the velocity of 8±0.6 µm/min measured previously [9] for N-terminally GST tagged KCBP (820–1261) containing both the dimerization domain at the N-terminus and the intact regulatory domain. Expressed and purified KCBP (820–1261) was unstable and tended to aggregate disallowing assay of the purified protein.

Thus, only the N-terminal dimerization domain is required for motility of KCBP. Deletion of the regulatory domain reduced, but did not abolish KCBP motility and did not affect the directionality of movement.

## Discussion

In this study, we have discovered a previously unsuspected self-association of KCBP into a dimer via its C-terminal regulatory domain. We have found that the negative coil of KCBP, a part of its regulatory domain, stabilizes KCBP dimers, which are formed by hydrophobic interactions between the residues of the calmodulin binding helices, another part of its regulatory domain. In our experiments, dimerization of KCBP via regulatory domain was observed both in crystals and in solution. In crystals, dimerization was observed for both *Arabidopsis* KCBP presented here and potato KCBP presented in our earlier works [12], [18] and re-analyzed here. In solution, *Arabidopsis* KCBP dimers were recognized using both size exclusion chromatography and analytical ultracentrifugation. Deletion of the regulatory calmodulin binding helix and the following negative coil destroyed the dimerization interface resulting in free KCBP monomers. Our crystal structure of *Arabidopsis* KCBP ruled out a possibility of the negative coil swapping between two neighbor molecules. Thus, the interactions of the negative coil with the microtubule-binding surface of the motor core do not contribute to the dimer interface. Although the negative coil is not a part of the dimerization interface, deletion of just the negative coil was, to our surprise, sufficient to break the KCBP dimers apart.

**Figure 6:**
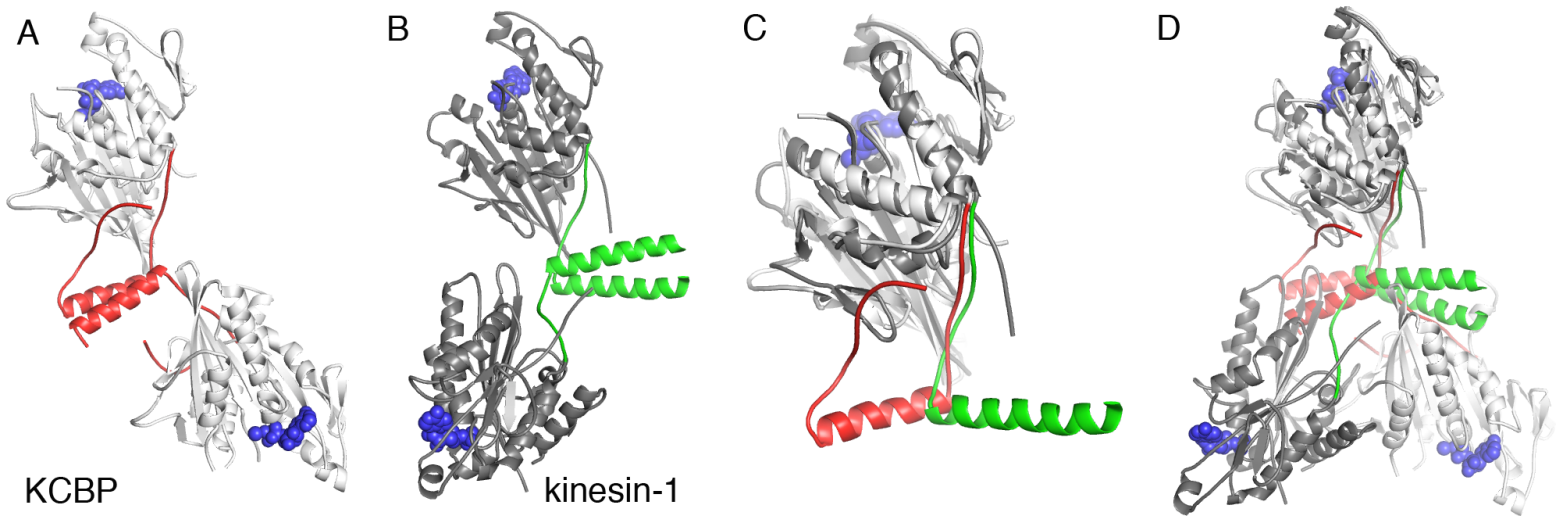
Structural similarity between KCBP and kinesin-1. *A-* Structure of Arabidopsis KCBP dimer. Color coding: grey – motor core, red – regulatory domain, blue – ADP. *B-* Structure of conventional human kinesin-1 (PDB ID 3KIN). Color coding: dark grey – motor core, green – neck, blue – ADP. *C-* Structural alignment of KCBP and kinesin-1 monomers. *D* – Structural alignment of KCBP and kinesin-1 dimers.

Another function of the regulatory domain of KCBP discovered here, namely dimerization, may have an evolutionary origin. As was noted previously, the linker connecting the regulatory helix to the motor core and carrying the name of neck mimic is strikingly similar by sequence and structure to the neck linker of kinesin-1 [12]. In kinesin-1, the neck linker is followed by a long helical dimerization domain that forms a coiled coil with a partner kinesin molecule [19]. The dimerization of kinesin-1 is supported by hydrophobic interactions within the coiled coil. Here we observe that the structural similarity between KCBP and kinesin-1 goes beyond the similarity of their motor heads and their neck/neck mimic linkers (Fig. 6). The helix following the neck mimic in KCBP, its regulatory helix, retains the ability to dimerize. The dimerization interface in KCBP is weaker than that in kinesin-1. Nevertheless, placing the negatively charged peptide, the negative coil, next to the dimerization interface, is required for KCBP’s ability to form dimers. Although the exact nature of dimer stabilization by the negative coil is still not clear, the described dimerization of KCBP indicates that evolutionarily speaking, KCBP is very close to the conventional kinesin-1.

Dimerization of KCBP via its regulatory domain was completely unexpected because its predicted dimerization domain is located on the opposite end of the polypeptide chain, N-terminal to the motor head. Having two distinct dimerization domains creates a possibility for KCBP to make continuous oligomeric structures. Two molecules of KCBP in the dimer formed via C-terminal helix are oriented such that their microtubule binding surfaces are near 90° relative to each other. This arrangement of KCBP molecules may be important for its physiological functions in orienting and bundling microtubules. In particular, KCBP is abundant in the plant-specific pre-prophase band and phragmoplast, and it functions in the formation and bundling of microtubules in these structures [20].

To establish the biological relevance of the regulatory helix self-association we performed microtubule bundling and motility assays. We found that deletion of the regulatory helix did not play a role in microtubule bundling and did not abolish motility of KCBP. The motor domain of KCBP by itself was sufficient to promote the microtubule bundling under the assay conditions of DIC. However, the structures of microtubule bundles formed by the KCBP motor domain by itself and by the KCBP motor plus regulatory domain may differ. Higher-resolution microscopy techniques would be required to resolve those differences. Low velocities demonstrated in motility assays by all tested constructs of KCBP indicate that this kinesin is likely involved in non-transport cellular events such as cytoskeleton organization. KCBP may function chiefly by aligning and bundling microtubules in a certain way.

Dimerization of KCBP via its regulatory domain brought into consideration another possible role for its negative regulators, KIC and calmodulin. Activated by Ca^2+^ ions, these Ca^2+^-binding proteins would bind to the regulatory helix of KCBP and break the dimers or higher order oligomeric structures if they do exist. Then, KCBP would be removed from microtubules in a complex with a regulatory protein.

In summary, we found that the negative coil of the regulatory domain is required for dimerization of KCBP via the regulatory domain. The dimerization interface formed by the regulatory helices is independent from the dimerization interface within the N-terminal domain of KCBP. We speculate that KCBP uses both dimerization interfaces either together or alternating them to support certain cytoskeletal structures.

## Supporting Information

Figure S1 **Analytical ultracentrifugation sedimentation equilibrium data for KCBP.** (A) KCBP (884–1244) and (B) KCBP (884–1253) were analyzed at three concentrations ranging from 5 to 10 µM at centrifugation speeds ranging between 3,000 rpm and 16,000 rpm at 20°C. Representative fits for each sample are shown. The solid red line shows the fit of the data to the ideal 1-component model, and the residuals of the fit are graphed to the right. The graphs were obtained using the program UltraScan3.
(JPG)

Movie S1 (AVI)

Movie S2 (AVI)
