# Supplementary figures and images for: Plant Kinesin-Like Calmodulin Binding Protein Employs Its Regulatory Domain for Dimerization
Source: PLoS One. 2013 Jun 21;8(6):e66669. doi: 10.1371/journal.pone.0066669 (PMC3689661; doi:10.1371/journal.pone.0066669)

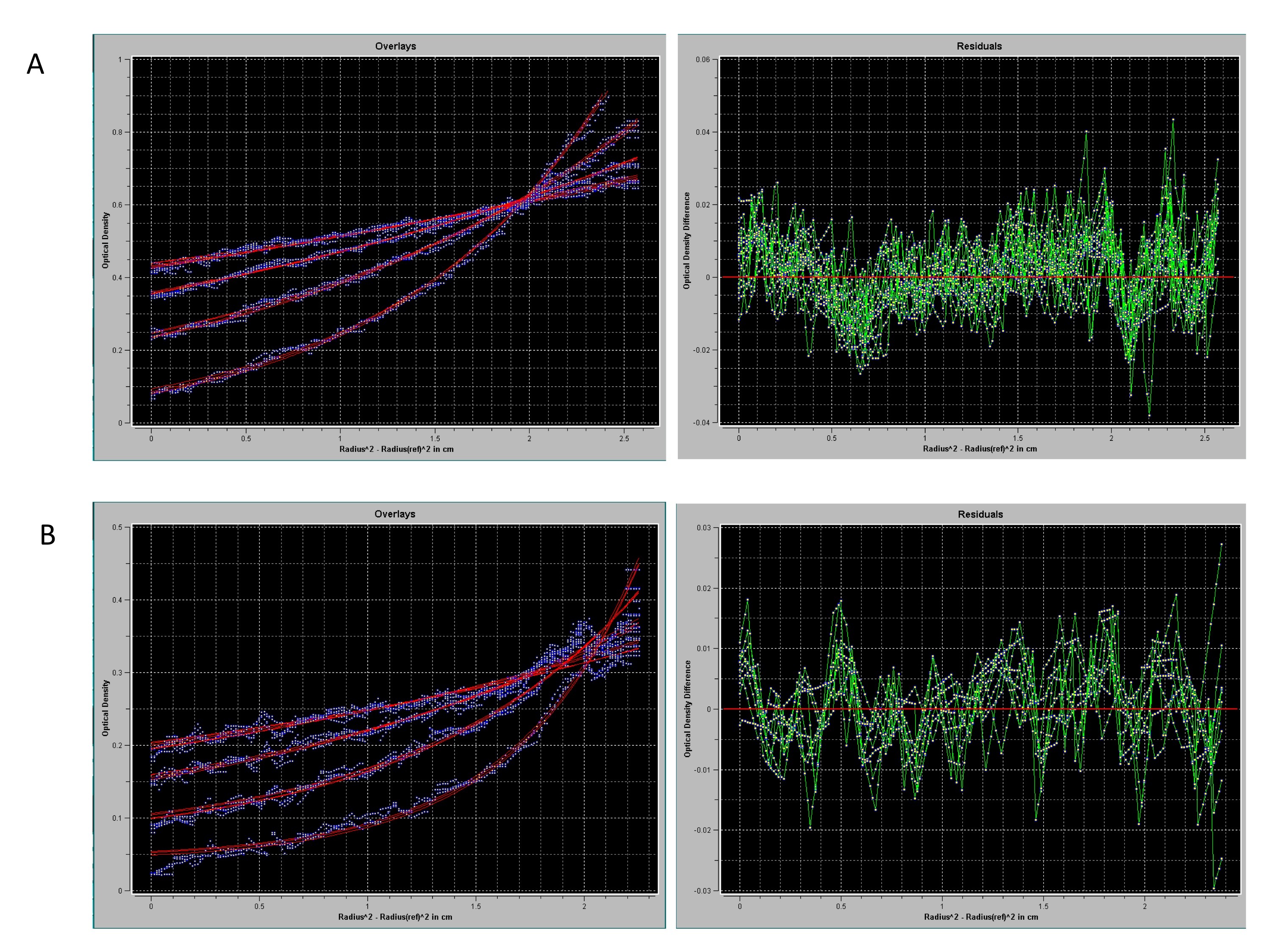

Supplement: Figure S1 — Analytical ultracentrifugation sedimentation equilibrium data for KCBP. (A) KCBP (884–1244) and (B) KCBP (884–1253) were analyzed at three concentrations ranging from 5 to 10 µM at centrifugation speeds ranging between 3,000 rpm and 16,000 rpm at 20°C. Representative fits for each sample are shown. The solid red line shows the fit of the data to the ideal 1-component model, and the residuals of the fit are graphed to the right. The graphs were obtained using the program UltraScan3. (JPG) [file pone.0066669.s001.jpg]
